# Effect of *PPAR-γ2* gene Pro12Ala and *ADR-β3* gene Trp64AArg polymorphism on glucose homeostasis in Type 2 diabetes subjects from Western India

**DOI:** 10.1186/1755-8166-7-S1-P101

**Published:** 2014-01-21

**Authors:** Ankna Shah, Frenny Sheth, Avisek Majumder, Bhavik Doshi, Navneet Shah, Premal Thakor, Rama Vaidya, Jayesh Sheth

**Affiliations:** 1 FRIGE’s Institute of Human Genetics, FRIGE House, Jodhpur Gam Road, Satellite, Ahmedabad-300015, India; 2 Department of Diabetes and Endocrinology; Sterling Hospital, Ahmedabad- 380052, India; 3 Gujarat Diabetic Association, Ahmedabad-380007. India; 4 Kasturba Health Society, Medical Research Centre, Mumbai-400056. India

## Background

Several studies have shown the effect of Pro12Ala polymorphism of *PPAR-γ2* on insulin sensitivity and Trp64Arg polymorphism in *ADR-β3* gene on obesity and insulin resistance in Type 2 Diabetic (T2D) subjects. The present study was carried out to find the interaction of these two gene polymorphisms and their combined effect on glucose homeostasis (HbA1C) in T2D subjects.

## Materials and methods

The present study comprises of 535 subjects (including 235 T2D & 300 controls). Genotyping was carried out for the above mentioned polymorphisms and glycosylated hemoglobin [HbA1C] levels were analyzed for each subject. All T2D subjects were divided into four groups according to their genotype. Group-I: 31 patients with Pro/Pro and Trp/Arg genotype; Group-II: 159 patients with Pro/Pro and Trp/Trp genotype; Group-III: 6 patients with Pro/Ala and Trp/Arg genotype; and Group-IV: 39 patients with Pro/Ala and Trp/Trp phenotype.

## Results

It was observed that 12Ala allele frequency was nearly equal in T2D patients and controls (9.0% vs. 9.1%, p>0.05). 64Arg allele frequency was 8.3% in T2D patients and 6.7% in controls (p>0.05). The mean HbA1C level was lower in T2D patients with 12Ala allele compared to patients homozygous for 12Pro allele (7.73±1.42% vs. 8.47±1.92%, p<0.02). However, no significant difference in mean HbA1C levels was observed in T2D patients with 64Arg allele compared to patients homozygous for 64Trp allele (8.50±1.81% vs. 8.31±1.88%, p>0.05). The mean HbA1C levels were higher in Group-I (8.62±1.84%, p<0.0092), Group-II (8.47±1.94%, p<0.001) and Group-III (8.26±1.71%, p>0.05) compared to grouPIV having a mean HbA1C of 7.65±1.37%.

## Conclusions

The protective effect of 12Ala allele is likely to be diminished in Group-III T2D patients in the presence of 64Arg allele. Polymorphisms of 64Arg and 12Pro alleles that is likely to play a role in controlling glucose homeostasis by gene-gene interactions.

